# Human relevant platforms for cutaneous wound healing research: current landscape, translational gaps, and emerging frontiers

**DOI:** 10.3389/fbioe.2026.1917429

**Published:** 2026-07-31

**Authors:** Fidha Latheef, K. Suthindhiran

**Affiliations:** Marine Biotechnology and Bioproducts Laboratory, Department of Bio-Medical Sciences, School of Bio Sciences and Technology, VIT, Vellore, Tamil Nadu, India

**Keywords:** bioprinting, cutaneous wound healing, *in vitro* wound models, skin-on-a-chip, three-dimensional skin equivalents, translational medicine

## Abstract

Cutaneous wound healing is a dynamic, multicellular process that unfolds across four interrelated phases - haemostasis, inflammation, proliferation, and remodellingeach governed by precise intercellular signalling that remains incompletely understood in its human context. Animal models and two-dimensional cell cultures have shaped much of what we know about wound biology, yet both consistently fall short when the question moves from mechanism to translation. They fail to capture the structural organisation of human skin, the particular rhythms of human immune activation and resolution, and above all the multifactorial pathology that makes chronic wounds-diabetic foot ulcers especially, so resistant to treatment. This review traces the development of human-relevant alternative models as a coherent scientific response to those failures: from scratch assays and monocultures through to three-dimensional reconstructed equivalents, organoid platforms, *ex vivo* tissue preparations, and skin-on-a-chip systems capable of dynamic perfusion and real-time wound monitoring. We assess each class of model not simply on its merits but on what specific biological gap it was designed to close and what gaps remain. Emerging analytical frameworks-multi-omics integration, spatial transcriptomics, microbiome and biofilm modelling, multi-organ-on-a-chip architectures, artificial intelligence, and neuro-immune crosstalk -are examined as the next Frontier. These findings show that no single platform will resolve the translational deficit; what is required is a deliberately combinatorial paradigm in which complementary systems are deployed in tiered sequence, each contributing the biological information it is best positioned to generate.

## Graphical Abstract

**Figure:**
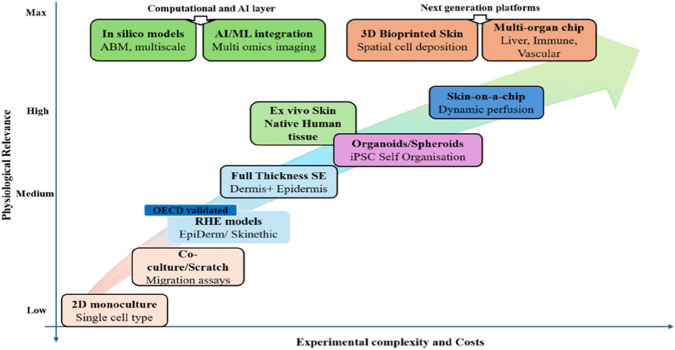
Graphical Abstract: Extended wound healing model complexity spectrum Alt text: Simple 2D cell culture; multi-organ, complex culture; computational models (in increasing order of physiological complexity) along a spectrum from left to right.

## 1 Introduction

The skin does not merely enclose the body,it actively mediates its relationship with a hostile external environment. It is the largest organ, which protects against pathogens, UV radiation, mechanical trauma, chemical insult, is a site of immune surveillance, thermoregulation, sensory integration, and maintains water homeostasis (Yousef et al., 2023). If that barrier is broken, the effects are more than just the wound itself; a lack of reliable healing is one of the most clinically significant biological shortcomings a patient can encounter and we are not yet as good at treating it as we are at studying it.

The distinction between acute and chronic wounds is not merely descriptive, it is mechanistic. Acute wounds arising from surgery, trauma, or thermal injury resolve through a coordinated reparative sequence in which each phase prepares the biological substrate for the next. Chronic wounds -diabetic foot ulcers, venous leg ulcers, pressure injuries were do not simply heal; they become arrested, locked in a state of non-resolving inflammation that actively prevents the proliferative and remodelling events on which tissue restoration depends. This issue is so significant that about 18 million new diabetic foot ulcer cases are registered worldwide annually (Armstrong et al., 2023) and most will be treatment refractory. These wounds reproduce the biological aspects of the wounds, impaired macrophage polarization, defective angiogenesis, dysregulated ECM remodelling, and persisting microbial colonization, least convincingly.

The healing process involves four stages that are interdependent: haemostasis, defended by platelet aggregation and fibrin clot formation; inflammation, characterised by the recruitment of neutrophils and macrophages; proliferation, driven by re-epithelialisation, granulation tissue deposition and neovascularisation; and remodelling is the final stage during which the collagen architecture matures and the scar tissue consolidates (Zhang et al., 2026). These phases overlap in time, and are controlled by feedback loops that are so complex that any disruption in any of them (as seen in diabetes, vascular disease, or immunosuppression) can upset the entire programme.

That scientific investigation carried on for decades by animal models has been all but accepted - and for good reason - as they provide whole-organism complexity that an *in vitro* system cannot match. The problem is not that animal data is uninformative; it is that it is not reliable enough to predict human outcomes, and that the disconnect is large enough to be a systemic scientific problem, not just a string of unfortunate missteps, in wound therapeutics. This gap is of biological origin, not just because rodents heal faster, but also because it affects drug response, inflammatory kinetics, and scar formation. This impact can’t be engineered away by studying them better.

This review aims to describe the range of alternative models that have been developed in response to that awareness that is human-relevant. Moving from 2D systems to next-generation microphysiological platforms, each section describes a different model class and the biological constraints that necessitated the creation of the next model. Section 5 offers a systematic comparative analysis, while Sections 6 and 7 focus on ethical, regulatory, and technical issues that have become relevant in the field of adoption. Section 8 explores emerging research frontiers. Finally, a multistage research approach that combines multiple methods is the most scientifically justifiable trajectory for future research.

## 2 Biological phases of wound healing and their molecular architecture

**FIGURE 1:**
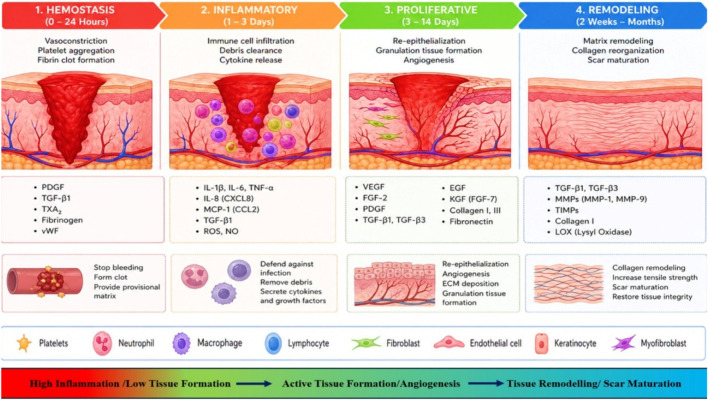
Illustrating different stages of wound healing with Molecular markers functioning in each stage.

Any serious evaluation of wound healing models must begin with a clear account of what those models are trying to replicate. The phases of wound repair are not simply sequential stages to be checked off; they are dynamically regulated, mutually dependent biological programmes whose execution requires precise intercellular communication across spatial and temporal gradients that most experimental systems do not come close to reproducing. Understanding where and how these programmes operate - and where they break down in chronic wound pathology - defines the biological requirements that alternative models must ultimately meet (See Figure 1).

### 2.1 Haemostasis

Haemostasis is the first response and possibly the first signal that follows. A reflex vasoconstriction that occurs when vascular injury occurs to limit the bleeding. Initiation of primary haemostasis by adhesion of platelets to subendothelial collagen and fibronectin, then a coagulation cascade that converts fibrinogen to fibrin creating a mechanically stable barrier (Kanji and Das, 2017; Sidonio et al., 2023). It is less well known that the first signal source for the repair process is also at this earliest moment of injury: the platelet. TGF-β, PDGF, VEGF, SDF-1/CXCL12 and endostatin are other cytokines released during degranulation which prep the microenvironment for the inflammatory and proliferative events that follow (Kirsner and Eaglstein, 1993). The apparent dispensability of platelets in wound repair, demonstrated by successful healing in platelet-depleted systems (Venter et al., 2016), highlights the redundancy of early wound-healing signals. This has likely been discussed an evolutionary advantage by ensuring robust wound closure, a complexity that reductionist *in vitro* models, which frequently exclude platelets, fail to capture. The activation of the complement cascade through C5 is another platelet degranulation-induced effect that links the haemostatic and inflammatory responses, and is not mentioned in models that focus on either of these response pathways alone (Abazari et al., 2022).

### 2.2 Inflammation

The inflammatory phase is where much of the pathological action in chronic wounds takes place, and where the divergence between animal models and human biology is most consequential. Vasodilatation and vascular permeability allow neutrophils-the first leukocytes to arrive which extravasate and use their antimicrobial capacity (phagocytosis, generation of reactive oxygen species, secretion of proteolytic enzymes) to quickly eliminate bacteria (Abazari et al., 2022). The issue is related to the dosage and the duration: neutrophils that remain longer than necessary become destroyers instead of defenders, and they cause collateral damage to the tissues; they send proteases to degrade the components of the ECM that are necessary for repair. This temporal dysregulation is a key part of the pathophysiology of chronic wounds, and is a feature that most static *in vitro* models would not be able to replicate (Berthiaume Fox et al., 2026; Joorabloo and Liu, 2022).

Macrophages are the central element of the inflammatory-to-reparative transition, and the quality of that transition determines whether a wound resolves or stagnates. The temporal switch from M1 to M2 function and secretion of cytokines that promote immune activation and pathogen clearance to M2 function and secretion of cytokines that allow fibroblast activation, ECM deposition and neovascularisation is made possible by the release of IL-10, VEGF and TGF-β (Gurtner et al., 2008). This transition is disrupted in diabetic wounds, with the M1 polarisation status remaining and M2 recruitment being delayed, while the wound environment continues to be pro-inflammatory beyond the time it should be pro-regenerative. That this M1-to-M2 switch can be pharmacologically redirected has made macrophage reprogramming one of the more actively pursued therapeutic strategies in the field -but modelling it requires systems that can sustain and phenotype immune cell populations over time, which excludes most conventional 2D cultures. Alongside macrophages, mast cells amplify early vascular responses through histamine and lipid mediators, whilst Langerhans cells and dermal dendritic cells link innate and adaptive immunity -cell populations that are almost entirely absent from engineered *in vitro* constructs.

### 2.3 Proliferation and remodelling

Re-epithelialisation begins within hours of injury, not days - a fact that is easy to lose sight of when studying cell migration in a scratch assay, where the biological context is stripped away. Wound-edge keratinocytes lose their contact inhibition, change the organization of their cytoskeletons, and migrate laterally across the provisional matrix, whereas basal keratinocytes and follicle-associated epithelial stem cells are stimulated to proliferate within the first 48–72 h after wounding in order to maintain the moving epithelial front (Singer and Clark, 1999; Shaw and Martin, 2009). This migration is driven by a calcium influx that activates cytoskeletal remodelling, amplified by KGF, IGF-1, and macrophage-derived nitric oxide (Barrientos et al., 2008), and regulated through mechano-transduction pathways, including Hippo-YAP -that respond to the physical properties of the wound substrate. That last point matters for model design: cells migrating on tissue culture plastic are receiving mechanosensory inputs that bear no resemblance to the compliant, dynamically remodelling wound bed they would encounter *in vivo*.

Neovascularisation is not incidental to healing; it is a rate-limiting step. Without adequate perfusion, the metabolic demands of the proliferating and remodelling wound cannot be met, and the result is the hypoxic, nutrient-deprived environment that characterises non-healing wounds. VEGF, PDGF, thrombin and bFGF activate existing endothelial cells to break down the basement membrane and grow towards angiogenic gradients, while nascent lumens develop into arterioles and venules stabilized by pericytes and smooth muscle cells (Eming et al., 2014). The contribution of BMC endothelial progenitor cells may be via vasculo-genesis although the significance of this mechanism in human wound healing is not fully understood (Rodrigues et al., 2019). In parallel, surrounding fibroblasts migrate into granulation tissue, deposit collagen I and III, fibronectin and glycosaminoglycans and secrete MMPs to remodel the provisional matrix (Gurtner et al., 2008). Under TGF-β/SMAD signalling a subpopulation becomes contractile myofibroblasts, which are responsible for wound contraction (Pakshir et al., 2020).

The remodelling phase; which may run for months to years, is where scar formation is determined. Progressive replacement of type III collagen by mechanically superior type I collagen, collagen cross-linking, and vascular regression all contribute to the maturation of scar tissue; the outcome, in humans, is rarely full structural restoration. Pathological remodelling - driven by unchecked TGF-β signalling, impaired macrophage egress, or defective MMP activity - produces either hypertrophic scar and keloid formation, or the atrophic, fragile tissue characteristic of chronic ulcers (Brem and Tomic-Canic, 2007; Falanga, 2005; Pakshir et al., 2020). These outcomes define the clinical problems that wound healing research most needs to solve -and they are the outcomes that current experimental models are least equipped to predict.

## 3 Animal models in wound healing research: contributions and constraints

**FIGURE 2:**
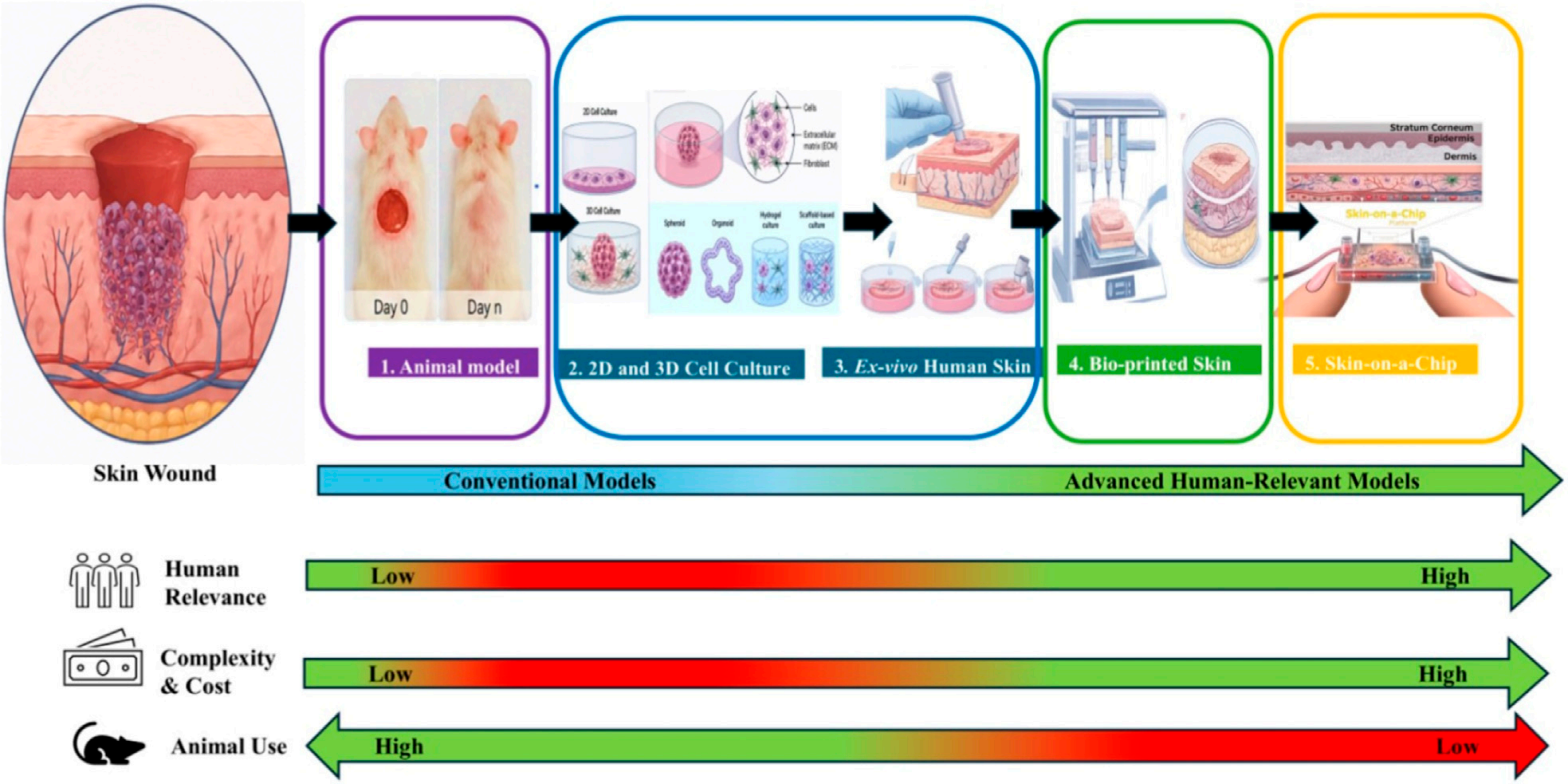
The image illustrates evolution of experimental model for cutaneous Wound healing Research.

It is important to grasp the reasons behind the development of alternative models, if not simply the fact that they were developed, then the specific biological dimensions that are failing animal models, which is where they are failing to be useful for the translational wound research. Animal system limitations are not simply practical constraints to be addressed by improved study design, but are intrinsic and inherent properties of animal biology that can not be eliminated by experimental manipulation. This is an evaluation of the value of animal models to the field, their differences from human physiology, and the importance of this difference in the clinical application of wound therapeutics (See Figure 2).

### 3.1 Preclinical animal models in wound healing research

Rodents, particularly mice and rats, are the model of choice for preclinical research in wound healing due to their availability, genetic manipulability and many methods. Common preparations are excisional and incisional wounds (for studies of acute wound healing), STZ induced diabetic rats, db/db leptin-receptor deficient mice (for impaired wound healing studies) and burn, ischaemic flap and infection models with *Staphylococcus aureus* and *Pseudomonas aeruginosa* (Phang et al., 2021). Targeted knockout strains -for MMP-9, TGF-β, VEGF, and others - have been invaluable for dissecting the contribution of specific molecular pathways to wound repair (Saeed and Martins-Green, 2023). Porcine models occupy a different position in the translational hierarchy: the structural and biochemical similarity of porcine to human skin in epidermal thickness, hair follicle density, and dermal collagen organisation which makes them the closest available whole-animal approximation of human wound healing, and they remain the standard for burn injury research, scar modelling, and advanced wound dressing evaluation (Rodrigues et al., 2022). The rabbit ear model is only suitable for research on hypertrophic scar. For most research programmes large animal models - canine, ovine, non-human primate - provide immunological and vascular fidelity that is close to human physiology, but are limited by ethical and regulatory issues combined with cost and complexity (Zindle et al., 2021). The practical consequence is that the most translationally relevant animal models are the least used, and the most widely used - rodents -are the least translationally relevant. That tension has shaped the field for decades.

### 3.2 Where rodent biology diverges from human wound repair

The most common anatomical difference between rodents and humans is the presence of the panniculus carnosus (striated muscle layer found under the skin) in rodents which plays a major role in wound closure by contracting the wound rather than through re-epithelialisation and ECM remodelling (Rodrigues et al., 2022). The clinical relevance of this difference goes beyond the academic realm: If the drug is responsible for the increased rate of wound healing in mice by increasing muscle tissue contraction, then a similar effect on muscle tissue is absent in humans. The signal is effective, but the prediction is not. This is exacerbated by the more frequent epidermal cell turnover in mice, which means that the timescales of healing are not at all synonymous with the longer courses seen in human patients, especially those with comorbidities (Zindle et al., 2021).

The immune differences are not as apparent but perhaps more significant. Inflammation and repair are closely linked in human wounds; the same cytokines that activate the immune system also activate fibroblasts to recruit them and stimulate angiogenesis. In ways that are hard to quantify, rodent immune responses differ from this in terms of cell type, cytokine balance, and kinetics of immune responses, such that inflammatory interventions tested in rodents have a poor predictive value (Rodrigues et al., 2022). Vascular architecture, collagen organisation, and scar formation phenotype differ as well - humans form hypertrophic scars and keloids that rodents do not, a fact that becomes clinically relevant the moment a wound care intervention is intended to modulate scarring. In many cases, although the structure of the porcine model is very similar to that of the human, it is not equivalent in expression of growth factors, enzyme activity, and intracellular signalling, and extrapolation cannot be easily made (Rodrigues et al., 2022).

### 3.3 The chronic wound problem

The challenge of translating animal models is particularly pronounced in the study of chronic wounds. In human beings, chronic wounds are not just prolonged acute wounds, but the result of chronic (often years of) systemic pathology: poorly controlled diabetes, progressive peripheral vascular disease, immunosenescence, chronic mechanical stress, microbial colonization that starts as transient contamination, but progresses to an antimicrobial-tolerant microbial biofilm. It is not just about creating hyperglycaemia and making a punch biopsy, it is about creating an environment that mimics the evolution of disease that normally takes years to occur - with organ systems not seen in rodents or seen differently (Takeo et al., 2015).

STZ-induced diabetic rats and db/db mice generate hyperglycaemia, but not peripheral neuropathy, not microvascular disease, not immune dysfunction of the kind that characterises clinical diabetes. Unlike human DFUs, they lack hypoxic gradients, dysfunctional macrophage populations and polymicrobial biofilms in the wounds (Zhang et al., 2026; Pendsey, 2010). When a treatment works in these models, the most that can be said is that it works in young, healthy, hyperglycaemic rodents with artificially induced skin defects - a biological context that overlaps only partially with the clinical target. The failure rate of preclinical to clinical translation is not just a matter of bad luck for wound therapeutics; it is due to a systematic mismatch between the models used to generate efficacy data and the patients likely to benefit.

### 3.4 Ethical and regulatory pressures

In most research jurisdictions, the 3Rs are no longer a goal, but a mandate, and the trajectory of the 3Rs is shifting from Replacement to Reduction and Refinement (Moran et al., 2016). As institutions have implemented increasingly rigorous protocols due to rigorously justifying the use of species selection, statistical power, and experimental endpoints, the demand is highest for the higher-order species - pigs, dogs, and primates, that have the greatest translational relevance (Tarantal et al., 2022). The irony is not yet completely unraveled: the ethical program to enhance animal welfare is effectively designed to encourage using the least translationally valid species, since studies on rodents are not as rigorously examined nor as expensive as studies on pigs or primates that have greater potential to provide predictive data.

Regulatory pathways for novel wound therapeutic approval require preclinical safety and efficacy data, and for now, animal data remains the standard. But the lack of standardisation across animal models and experimental protocols - in species, wound induction method, outcome measures, and follow-up duration, means that the animal evidence base is often less coherent than it appears, complicating regulatory submissions and contributing to the reproducibility problems that have drawn increasing scrutiny across biomedical research (Saeed and Martins-Green, 2023). These pressures the scientific, ethical, and regulatory which simultaneously have created both the need and the institutional space for human-relevant alternative platforms. The sections that follow examine what those platforms currently offer, and what they do not yet provide.

## 4 Human-relevant alternative models for wound healing studies

The scientific case for alternative models is not simply that animal models are ethically problematic or economically inefficient - though both are true. It is that the specific biological questions most pressing in wound healing research cannot be reliably answered from animal systems and that a generation of experimental systems have been created to fill this gap. It is followed here, not as a list of model tools, but as a scientific argument; a list of what each model does is not as significant as the biological argument for the need for each (see Table 1).

**TABLE 1:** Comprehensive comparative analysis of human-relevant alternative models for cutaneous wound healing research.

Category	Model type	Physiology/Pathology simulated	Model construction	Wound induction method	Key findings/Readouts	Translational relevance	Limitations	References(s)
2D *in vitro*	Monoculture (fibroblasts/keratinocytes)	Re-epithelialisation; ECM deposition; collagen synthesis; wound contraction	Single cell type on flat TCP/glass; fibroblasts (L929, HDFs) or keratinocytes (HaCaT, primary)	Scratch wound assay; mechanical disruption of confluent monolayer	Cell-specific responses to growth factors, cytokines, nanomaterials; migration/proliferation kinetics	High-throughput primary screening; preliminary mechanistic studies; low cost	Lacks 3D architecture, ECM, multicellular crosstalk, immune components; altered morphology/gene expression vs. *in vivo*	Grada et al. (2017), Pastar et al. (2014)
2D *in vitro*	Co-culture (fibroblast–keratinocyte)	Dermal–epidermal paracrine signalling; coordinated migration; cytokine exchange	Transwell or direct co-culture of HDFs and keratinocytes on flat substrates	Scratch assay; transwell migration assay	Paracrine growth factor exchange; coordinated wound closure kinetics; inter-layer cytokine signalling	Improved intercellular communication modelling vs. monoculture; accessible format	No 3D structure; absent vascularisation and immune cells; dual-culture condition optimisation is challenging	Pastar et al. (2014), Rodrigues et al. (2019)
3D reconstructed skin	Reconstructed human epidermis (RHE)	Epidermal stratification; stratum corneum formation; barrier function; cytotoxicity	Keratinocytes at air–liquid interface on inert supports; commercial: EpiDerm, EpiSkin, SkinEthic	Topical chemical/mechanical disruption of stratum corneum	Barrier function assessment; transdermal drug penetration; irritation/corrosion (OECD TG 431/439 validated)	OECD-validated regulatory replacement for draize testing; high batch-to-batch reproducibility; widely used in cosmetic/pharma safety	No dermal compartment, fibroblasts, immune cells, or vascularisation; cannot model angiogenesis, ECM remodelling, or chronic wounds	Netzlaff et al. (2005), Mathes et al., 2014
3D reconstructed skin	Full-thickness skin equivalent (FTSE)	Dermal–epidermal interactions; re-epithelialisation; ECM remodelling; collagen deposition; chronic inflammation	Fibroblast-populated collagen/fibrin dermal scaffold with stratified keratinocyte epidermis; optional macrophages/dendritic cells	Punch biopsy; scalpel excision; UV irradiation	Bidirectional fibroblast–keratinocyte signalling; MMP activity; collagen remodelling; inflammatory cytokine dynamics; biomaterial evaluation	Closest engineered construct to native skin; supports evaluation of novel dressings, hydrogels, nanoparticle therapeutics	No functional vascularisation (diffusion-limited); complex, costly fabrication; variable reproducibility across scaffold/cell sources	Bell et al. (1981), El-Ghalbzouri et al. (2002), Mathes et al. (2014)
*Ex vivo*	Human skin explant	Acute and burn wound healing; barrier disruption; inflammatory response; host–pathogen interactions; biofilm formation	Surgical discard skin (abdominoplasty, mastectomy) maintained at air–liquid interface; native architecture preserved	Punch biopsy; scalpel excision; laser ablation; burn induction	Re-epithelialisation; granulation tissue formation; ECM deposition; immune cell activation (langerhans cells, dermal macrophages); antimicrobial dressing evaluation	Highest physiological fidelity among non-circulatory systems; immune-competent; retains skin appendages; ethically accessible human tissue	Limited viability (days–weeks); absent blood circulation, endocrine signalling, circulating immune recruitment; donor variability (age, health, site)	Wurbs et al. (2024), Chen et al. (2024)
Organoid/spheroid	Skin organoids (iPSC/adult stem cells)	Epidermal morphogenesis; stem cell niche biology; skin appendage formation; regenerative signalling	iPSC or adult progenitor cells self-organised in matrigel or suspension; forms stratified epidermis, dermal compartment, hair follicles	Micro-wound induction by mechanical disruption or laser	Stem cell proliferation, differentiation, migration; wnt/β-catenin, TGF-β, notch signalling; appendage regeneration; regenerative therapy assessment	Recapitulates developmental programmes; suitable for patient-specific modelling; no external scaffolding required	Incomplete vascularisation; lacks functional immune compartment; stochastic self-organisation reduces reproducibility; complex, costly, slow	Lee et al. (2020), Fatehullah et al. (2016)
Organoid/spheroid	MSC spheroids	Paracrine-mediated wound repair; angiogenesis; anti-inflammatory signalling; collagen synthesis	MSCs aggregated by hanging drop or ultra-low-adhesion culture; mono- or co-culture with endothelial/immune cells	Indirect: Spheroid-conditioned medium or co-culture with wounded monolayer	Enhanced VEGF, HGF, TGF-β, IL-10, extracellular vesicle secretion vs. 2D culture; pro-angiogenic and anti-inflammatory effects	Scalable cell therapy testing platform; superior paracrine activity vs. 2D MSC cultures; evaluates stem cell-based wound therapeutics	No systemic immune context; limited tissue complexity; variable secretory profile between donors; no mechanical stimulation	Bartosh et al. (2010), Fennema et al. (2013)
Micro physiological	Skin-on-a-chip (SoC)	Dynamic wound repair; re-epithelialisation; angiogenesis; chronic/diabetic wound pathology; drug delivery	Multilayer PDMS microfluidic device: Keratinocyte epidermis/porous membrane/fibroblast dermis/endothelial vascular channel; continuous perfusion	Mechanical scratch; chemical wounding; laser ablation on-chip	Real-time monitoring of migration, angiogenesis, matrix deposition; modelling of hyperglycaemia, hypoxia, inflammatory milieu; dynamic drug delivery	Most physiologically dynamic engineered platform; enables perfusion, mechanical stimulation, disease-specific microenvironments; reduces animal dependency	High technical complexity/cost; absent standardised protocols; limited immune compartment; low throughput; specialist infrastructure required	Bhatia and Ingber, (2014), Wufuer et al. (2016), Ramadan and Ting, (2016)
Micro physiological	3D bioprinted skin constructs	Spatially organised wound repair; full-thickness wound coverage; chronic wound treatment evaluation	Extrusion, inkjet, or laser-assisted bioprinting of cell-laden bioinks (collagen, fibrin, GelMA) in defined geometries; multi-nozzle for layered skin	Defined wound geometry created during fabrication; subsequent real-time monitoring	Spatial organisation of keratinocytes, fibroblasts, endothelial cells; ECM architecture; vascular channel printing; bioactive dressing evaluation	Enables patient-specific wound geometry and cellular architecture; scalable construct fabrication; integrates with SoC platforms	Print resolution limits cell viability; lacks innervation and immune cells; requires specialist equipment; regulatory framework nascent	Vijayavenkataraman et al. (2021), Paek et al. (2024)
Micro physiological	Multi-organ-on-a-chip (MoC)	Systemic pharmacokinetics; hepatic drug metabolism; immune trafficking; metabolic disease effects on wound healing	Skin SoC connected to liver, kidney, immune, and vascular modules via shared microfluidic circuit	Wound induction in skin module; drug administered through systemic circuit	Transdermal drug absorption and hepatic metabolism; systemic cytokine effects on wound repair; diabetic multi-organ pathology modelling	Only platform able to model systemic influences on wound healing; supports evaluation of systemically delivered therapeutics	Extremely high complexity; inter-module crosstalk difficult to isolate; no standardised architecture; early-stage regulatory status	Bhatia and Ingber (2014)
Computational	In silico/computational models	Multiphase wound healing dynamics; cytokine gradient formation; angiogenesis; chronic wound pathology; drug release kinetics	Mathematical equations, agent-based modelling (ABM), finite element analysis, multiscale frameworks parameterised with experimental data	Simulated injury parameters (wound geometry, cell density, cytokine concentration)	Identification of critical regulatory thresholds; prediction of drug intervention effects; personalised healing trajectory modelling; optimisation of *in vitro* experimental design	Complements all experimental platforms; low cost; no ethical constraints; supports precision medicine and mechanistic hypothesis generation	Dependent on quality of experimental parameterisation data; biological simplifications limit fidelity; not a standalone replacement for experimental validation	Sherratt and Murray, (1990), Flegg et al. (2015)

The table depicts Comprehensive comparative analysis of human-relevant alternative models from 2D to Next-generation models for cutaneous wound healing research.

### 4.1 Two-dimensional in vitro cell culture models

Two-dimensional cell culture is where most mechanistic wound healing research begins, and for good reason. When the goal is high-throughput screening or preliminary testing of hypotheses, seeding the dermal fibroblasts or epidermal keratinocytes under defined conditions on flat plastic/glass surfaces that are reproducible, scalable, and inexpensive (Grada et al., 2017; Rodrigues et al., 2019). The fibroblast monoculture has been a source of many of the insights we have into collagen synthesis kinetics, MMP secretion profiles and molecular responses to TGF-β stimulation. Keratinocyte monocultures provided a platform for initial characterisation of re-epithelialisation signal transduction, barrier protein expression and growth factor dependency of epidermal regeneration (Li et al., 2007; Pastar et al., 2014). That is the real knowledge that is valuable.

There is an equally real limitation: there is no single-cell-based data to replace the emergent biology that can only be generated through the interactions of multiple cell types in an *in vivo* setting when dealing with wound healing. Co-culture systems were developed to partially address this-fibroblast-keratinocyte preparations in transwell or direct contact formats enabled the study of paracrine signalling, growth factor exchange, and coordinated migratory responses that monocultures cannot generate (Pastar et al., 2014). Compared with monocultures, these systems offer improved insight into intercellular communication and tissue behaviour, yet they remain considerably less representative than the integrated complexity of *in vivo* systems. Vascularisation, immune, three-dimensional ECM architecture is absent, and the mechanical environment bears no resemblance to the compliant, dynamically remodelling wound bed *in vivo* (Rodrigues et al., 2019).

This paradox is well exemplified by the scratch wound assay which is the workhorse of the 2D wound biology paradigm. Its use is widespread because is simple, reproducible and allows to obtain measurable data of cell migration and closure kinetics with low infrastructure. But in fact, it measures the reaction of a single layer of cells on stiff plastic when they’re subjected to a mechanical “scratch” - a model of wound closure in roughly the same way that a weather vane represents atmospheric dynamics. Mechanical disruption causes uneven damage to the cells and substratum; the assay cannot distinguish migration from proliferation by simply inhibiting the drugs; all the process takes place in the biochemical and mechanical environment which is different from that in any tissue (Kramer et al., 2013; Liang et al., 2007). These are not flaws that make the assay invalid, but are reasons to be careful about what it can say, and not what it cannot (Duval et al., 2017; Rodrigues et al., 2019). The field moved on from these limitations, as it had to.

### 4.2 Three-dimensional in vitro skin models

The recognition that planar geometry and monocellularity were not incidental features of 2D systems but fundamental constraints on the biology they could represent drove the development of three-dimensional constructs in which cells are organised within an ECM scaffold that begins to approximate the spatial architecture of real tissue. This was a conceptual shift, not merely a technical one -it repositioned the *in vitro* wound healing model from a single-cell readout system to a tissue analogue with at least some of the structural features that make wound biology what it is.

#### 4.2.1 Reconstructed human epidermis

Reconstructed human epidermis models are among the few alternative systems to have achieved genuine regulatory traction. By culturing keratinocytes at an air-liquid interface, these constructs develop a stratified multilayered epidermis with a functional stratum corneum whose barrier properties are sufficiently close to native human skin to satisfy OECD Test Guidelines 431 and 439 for skin irritation and corrosion testing - a validation milestone that established, in principle, that *in vitro* skin models could generate data sufficient for regulatory decision-making (Netzlaff et al., 2005; El-Ghalbzouri et al., 2002). Commercial systems - EpiDerm, EpiSkin, SkinEthic - are standardised, batch-consistent, and widely adopted in cosmetic and pharmaceutical safety assessment (Mathes et al., 2014). Their principal scientific contribution has been in barrier function research, topical drug permeation studies, and safety screening for topical wound therapeutics.

Their principal scientific limitation is structural and cannot be resolved without changing what the model is: there is no dermis. Without fibroblasts, there is no ECM remodelling; without immune cells, there is no inflammatory signalling; without vasculature, there is no angiogenesis. For epidermal-level questions, RHE models are fit for purpose and well-validated. For wound healing beyond the epidermis - which is to say, for most of the biology that matters clinically - they are the wrong tool. The field needed a dermal compartment.

#### 4.2.2 Full-thickness skin equivalents

Full-thickness skin equivalents addressed that need by combining a fibroblast-populated collagen or fibrin dermis with a stratified keratinocyte epidermis, enabling -for the first time in an engineered construct - the study of bidirectional dermal-epidermal communication (Bell et al., 1981; El-Ghalbzouri et al., 2002). The spectrum of wound healing questions that can be addressed by these models is much wider: MMP activity, collagen deposition kinetics, fibroblast keratinocyte paracrine signalling, ECM remodelling dynamics, and the impact of candidate wound therapeutics on tissue-level rather than single-cell outcomes (Rodrigues et al., 2019; Pastar et al., 2014). More complex ones include macrophages and dendritic cells, so at least some aspects of inflammatory signal transduction pathways, which are conspicuously missing from simpler systems, can be modelled (Maltman and Przyborski, 2010). Full-thickness equivalents provide a much more informative, 3-D format for biomaterial and drug delivery evaluation, including evaluation for -hydrogels, nanoparticle dressings, bioactive scaffolds - with drug penetration and retention data that flat monolayers simply cannot (Mathes et al., 2014).

The residual limitation is vascularisation. Without a perfusable microvascular network, nutrient and oxygen delivery depends on passive diffusion - a constraint that limits construct viability and precludes the study of angiogenic responses that are integral to the proliferative phase of healing. Some groups have incorporated microvascular elements, but none has yet achieved the haemodynamic complexity and physiological responsiveness of native vasculature. The cost and fabrication complexity of these models also introduce practical barriers; variability in scaffold materials and cell source characterisation affects reproducibility in ways that standardised protocols have not yet fully resolved (Abaci et al., 2016; Mathes et al., 2014). These limitations pointed toward a different class of model entirely - one derived not from engineering but from the tissue itself.

### 4.3 *Ex vivo* human skin models

*Ex vivo* skin models occupy a unique position in the alternative model landscape precisely because they do not attempt to engineer a tissue analogue - they use the actual tissue. Freshly excised human skin from surgical discard after abdominoplasty, mastectomy and elective procedures is available that is freshly removed and maintained at an air liquid interface with well-defined conditions, allowing for a period of experimentation long enough to allow for the maintenance of its native architecture, cellular composition and biochemical environment (Wurbs et al., 2024). The biological fidelity this confers is qualitatively different from anything achievable in an engineered construct: resident immune populations -Langerhans cells, dermal macrophages, mast cells - are present and functional; skin appendages including hair follicles and sebaceous glands are intact; the ECM has its native composition and spatial organisation rather than a laboratory approximation of it.

For wound healing research, this matters in ways that are not marginal. The immunological competence of *ex vivo* skin and its capacity to mount genuine inflammatory responses mediated by resident immune cells rather than artificially supplemented ones makes it the most physiologically realistic system currently available for studying the early phases of wound repair, host-pathogen interactions, and the assessment of wound dressings and antimicrobial agents under conditions that approximate clinical tissue (Chen et al., 2024). Topical antimicrobials that have exhibited greater correlation with clinical outcome than their reconstructed equivalents have been evaluated using *ex vivo* burn wound models (Chen et al., 2024). *Ex vivo* human skin explants maintain the native epidermal-dermal architecture and provide a more physiologically accurate model of wound-healing dynamics than reconstructed skin equivalents, which lack this structural complexity (Guo et al., 2025). A key advantage that no engineered construct offers is the ability to investigate the dynamics of biofilm and host-pathogen interactions, two critical aspects of chronic wound recalcitrance in a human tissue context. The limitations are time and system. Excised tissues cannot be maintained for an extended period of time - days to a few weeks, depending on the conditions for culture and the quality of the tissue that long enough to address many experimental questions but not the months-long evolution of a non-healing wound. Since peripheral immune cells are not recruited in the absence of circulation, neither is endocrine signalling, nor is there any dynamic response to changes in perfusion, all of which contribute to the *in vivo* wound environment in ways that cannot be reproduced in *ex vivo* models. Experimental design can account for donor variability (age, comorbidities, anatomical location, medication history, and microbiome composition) that can introduce heterogeneity (Chen et al., 2024; Wurbs et al., 2024). These do not prevent their use, but they do restrict the types of questions that *ex vivo* models are appropriate for. The field needed a different way for dynamic, long-term and perfusion dependent studies.

### 4.4 Organoid and spheroid models

Organoid technology represents a conceptually distinct approach from both engineered constructs and *ex vivo* preparations. Rather than assembling a tissue from components or preserving one that already exists, organoid systems exploit the intrinsic developmental programming of stem cells -their capacity, under appropriate conditions, to self-organise into structures that recapitulate tissue architecture without external scaffolding. In advanced preparations, iPSC-derived or adult-progenitor-cell-derived skin organoids can form stratified epidermis, a dermal compartment, and - in advanced preparations - hair follicles and sebaceous glands, reflecting morphogenetic programmes which no scaffold-based construct has yet replicated (Lee et al., 2020; Abaci et al., 2016). The ability to form appendages is also a scientific finding with implications: hair follicles are not just features -they are also stem cell niches and re-epithelialisation reservoirs for wound healing that have not been well modeled in engineered systems without hair follicles.

Organoids provide a unique opportunity for studying stem cell biology which is not available in other platforms, especially in the context of wound healing research. In a self-organised three-dimensional tissue, the physiological relevance of the tissue cannot be attained by either monolayer culture or scaffold construct, allowing the interrogation of signalling pathways involved in tissue regeneration such as Wnt/β-catenin, TGF-β, and Notch (Lim and Nusse, 2013). Spheroids as less complex three-dimensional aggregates complement this by offering tractable systems to examine paracrine interactions, wound responses and, in the case of MSC spheroids, the increased secretory activity which makes stem cell-based wound therapy a viable therapeutic approach. MSC spheroids produce significantly higher levels of VEGF, HGF and anti-inflammatory cytokines than 2D MSC (Bartosh et al., 2010), both for mechanistic study and therapeutic testing.

The restrictions are clearly known and fair: lack of vascularization, lack of immune compartment, stochastic self-organisation that leads to heterogeneity among preparations and technical and cost requirements of iPSC culture. They are not minor hurdles, and because organoids created in different labs can be different from one another (due to differences in production protocols), this is a reproducibility issue which the field is still working to resolve (Kim et al., 2020; Rossi et al., 2018; Fatehullah et al., 2016). Despite their potential, organoids are still static systems, and static systems have a basic limitation: they can’t mimic the dynamic, mechanically active, perfused environment of a wound in a living tissue. The ceiling was a whole other engineering challenge to achieve.

### 4.5 Microphysiological systems and skin-on-a-chip

Skin-on-a-chip platforms were developed from a specific insight: that the most biologically significant features missing from all static *in vitro* systems-perfusion, mechanical stimulation, dynamic oxygen and nutrient gradients, the continuous influence of flowing fluid on cellular behaviour - are not details that can be added to a static system but are architectural requirements that necessitate a different design philosophy altogether (Bhatia and Ingber, 2014; Wufuer et al., 2016). To overcome this, the concept of engineering cellular microenvironments has emerged at the scale at which tissue biology is actually playing out - multilayered channels filled with keratinocytes, fibroblasts and endothelial cells that are separated by porous membranes and linked to fluid circuits that emulate interstitial and vascular flow (see Table 2). This is not just a more advanced culture-based system, but a qualitatively new experimental system in which cells receive dynamic cells signals - shear stress, morphogen gradients, removal of metabolic wastes - that dictate the cells behaviours *in vivo* (Ramadan and Ting, 2016; Wufuer et al., 2016).

**TABLE 2:** Advanced micro-engineered and skin-on-a-chip platforms in wound healing.

Model/System	Key engineering features	Biological components	Functional outcomes	Relevance to wound healing	References
Microfluidic skin-on-a-chip	Perfusion flow; layered microchannels	Keratinocytes, fibroblasts	Simulates inflammation, edema	Models wound inflammation and drug response	Renò et al. (2026)
Vascularised skin models	Microvascular networks within ECM	Endothelial cells + skin cells	Perfusable vasculature formation	Critical for angiogenesis studies	Renò et al. (2026)
Microfluidic wound-healing assay chips	Controlled injury zones in chip	Epithelial cells	Quantitative migration and closure kinetics	Drug screening and healing dynamics	Renò et al. (2026)
Immune-integrated skin-on-chip	Addition of immune cells + cytokine control	Keratinocytes, fibroblasts, immune cells	Simulates immune response	Models chronic wounds and infection	Jeong et al. (2023)
Multi-organ-on-a-chip systems	Skin connected with liver/immune modules	Multi-tissue integration	Systemic drug metabolism studies	Important for transdermal pharmacokinetics	Jeong et al. (2023)
3D bioprinted wounded skin models	Spatial deposition of cells + biomaterials	Multi-layered skin + ECM	Recreates wound microenvironment	Personalised wound models (diabetic, chronic)	Ahn et al. (2023)
Pumpless microfluidic skin chips	Gravity-driven flow systems	Epidermal + dermal layers + vasculature	Long-term culture stability	Reduces complexity, improves usability	Mohamadali et al. (2023)

The Table depicts Advanced Micro-engineered and Skin-on-a-Chip Platforms with key features, biological components, functional outcomes and its relevance in wound healing.

The implications for the study of wound healing are significant. Fidelity of diabetic wound pathology in this model is not matched by other models, as all three disease-specific microenvironments -sustained hyperglycaemia, controlled hypoxia, defined inflammatory cytokine profiles thatcan be introduced to the model in a reproducible and tunable manner; this allows for mechanistic investigation of diabetic wound pathology that cannot be achieved in rodent models or static *in vitro* systems (Phang et al., 2021; Zhao et al., 2024). High resolution imaging of wounds induced on-chip can be used to obtain real time information on migration, matrix deposition and angiogenic sprouting, which are not available in endpoint-only systems. Drug efficacy data can be acquired with meaningfully greater *in vivo* predictive relevance than static assays, with the aid of therapeutic agents, such as nanoparticulate formulations, growth factor cocktails and hydrogel matrices, which can be administered through the microfluidic circuit under conditions that mimic the pharmacokinetic environment in the wound bed (Bhatia and Ingber, 2014; Wufuer et al., 2016).

The honest assessment is that SoC systems remain technically demanding and expensive, require specialist microfabrication infrastructure, and lack the standardized protocols that would enable consistent inter-laboratory comparison. The immune compartment, though expanding with recent systems have incorporated monocyte-derived macrophages and neutrophil migration that still falls short of the immunological complexity of native tissue. And the throughput achievable on current platforms, though improving, does not support the kind of large-scale compound screening for which 2D systems remain the practical choice. These are challenges being actively addressed through advances in microfabrication, bioprinting, and laboratory automation; the trajectory is clearly toward broader accessibility. What SoC systems offer now, even in their current form, is the most physiologically dynamic engineered wound healing platform available. That capability, for the questions that matter most in translational research, is not replicated anywhere else.

### 4.6 In silico models as complementary tools

Computational modelling has a unique place in wound healing research, not so much as a tool to observe biology *in situ*, but as a tool to systematically think about it. Biological processes can be captured in a mathematical model, an agent-based simulation, and/or a multiscale computational framework that can formalise complexity and uncover regulatory logic that is not apparent from experiments, which would otherwise require years and a significant number of resources to test (Sherratt and Murray, 1990; Flegg et al., 2015). Initial reaction-diffusion models formally described wound closure dynamics; agent-based models examined cell behaviors and their emergence at the macroscale in the healing of tissues; and recent multiscale models have sought to bridge the gap between molecular events to tissue-level healing dynamics at multiple scales of biology (Faglia et al., 2010; An, 2008).

The best use of *in silico* models is not as a predictive tool, but as an analytical companion to an experimental system. Computational models can be parameterised with quantitative data derived from the scratch assay, 3D skin equivalents, and SoC platforms, leading to enhanced biological relevance and predictive power of the models, migration velocities, secretion rates of cytokines, and dynamics of gene expression. Model outputs, on the other hand, can help determine the experimental conditions best suited to provide mechanistically meaningful results, thereby minimizing empirical effort in the wet lab (Siepmann and Peppas, 2011). For chronic wound and diabetic wound modelling specifically, *in silico* approaches offer the possibility of systematically exploring how different combinations of pathological inputs - hyperglycaemia, hypoxia, altered macrophage function, reduced perfusion which interact to produce the range of clinical phenotypes observed, a question that no single experimental system can address (Flegg et al., 2015). The accuracy of these models depends fundamentally on the quality of the experimental data used to build them, and biological simplifications are unavoidable. Yet as machine learning and systems biology methods continue to mature, the gap between computational prediction and biological reality is narrowing, making *in silico* tools increasingly indispensable.

## 5 Comparative analysis of alternative models

**FIGURE 3:**
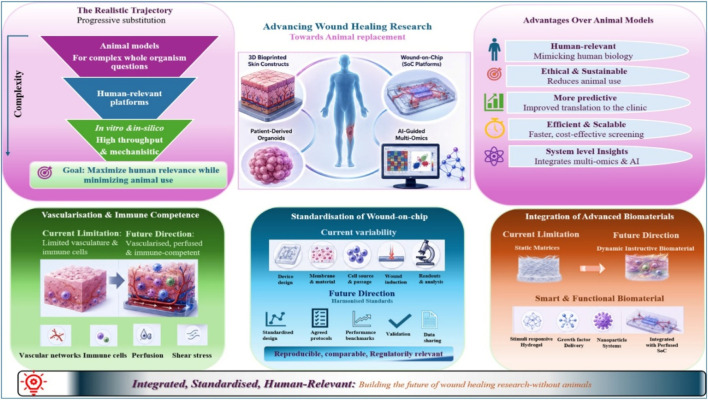
Illustrating standardised human relevant future of wound healing research without animals.

Having traced the development of each model class and the biological rationale underpinning it, a direct comparative assessment is warranted. The goal here is not to rank these systems in a single hierarchy of merit that framing misrepresents, how they should be used, but to map the dimensions along which they differ, so that model selection can be driven by the scientific question rather than by familiarity or convenience (See Figure 3).

### 5.1 Physiological relevance

There is a physiological fidelity gradient, but not linear, between these platforms. At the opposite end of the spectrum are 2D monocultures, consisting of single cell types on rigid plastic, devoid of ECM architecture, spatial organization and multicellular context (Edmondson et al., 2014; Mathes et al., 2014). Three-dimensional skin equivalents do better than these, by adding the interactions between cells, but without adding immune cells or functional blood vessels, they remain lump than is needed for most wound healing questions (El-Ghalbzouri et al., 2002; Mathes et al., 2014). *Ex vivo* preparations are a qualitative leap which not due to their higher performance on the same metrics as engineered constructs, but because it introduces biological characteristics that have not yet been replicated in engineering: resident immune competence, native ECM biochemistry, intact appendage structures (Wurbs et al., 2024). Self-organised developmental complexity (organoids) and dynamic perfusion and mechanical stimulation (SoC platforms). None of these individually offers a comprehensive systemic perspective (recruitment of circulating immune cells, hormonal signalling, multi-organ metabolic interactions) that governs wound healing in the living organism. That observation is not a counsel of despair; it is the argument for a combinatorial approach.

### 5.2 Experimental complexity and cost

The practical demands of these systems scale, roughly but not perfectly, with their physiological complexity. The 2-D cultures, which need standard cell biology infrastructure, can provide rapid and inexpensive data and can be easily adopted for high throughput formats and used at the screening phase of therapeutic development (Edmondson et al., 2014). 3D models require more: scaffold preparation, multi-step protocols of differentiation, longer culture durations, and more, but are still accessible to a good cell biology lab (Mathes et al., 2014). *Ex vivo* preparations add the logistical problems of securing tissue, sterile preparation, and coordinating donors, as well as the experimental design problems of variability among donors. The presence of iPSC culture expertise is required for organoid systems and extended time scales which are not practical for large-scale screening. SoC platforms demand access to microfabrication facilities and fluidic control systems that remain concentrated in specialist engineering environments, though this is changing as standardised device formats become commercially available (Ronaldson-Bouchard and Vunjak-Novakovic, 2018). The practical implication is that experimental complexity should be matched to the biological question: using a SoC platform to answer a question that a scratch assay could address is not rigour, it is misdirected resource.

### 5.3 Regulatory acceptance

Regulatory acceptance is not necessarily a measure of the maturity of the validation, but that is a point that should be emphasised as it is the most sophisticated physiologically that are not necessarily the most accepted by the regulators. Two-dimensional systems facilitate exploratory research, but have little regulatory power. The point is, there is a route to regulatory acceptance of alternative testing methods and it is passable: RHE models, in particular EpiDerm and EpiSkin, are now fully validated for OECD skin irritation and skin corrosion testing and are already used in cosmetic and pharmaceutical regulatory submissions (Marx et al., 2020). It is only recently that *ex vivo* and microphysiological preparations have attracted the attention of regulatory authorities, although the lack of harmonization means their use is not yet standardized. Scientifically compelling but not yet established to meet regulatory evidentiary standards, SoC and organoid platforms remain in early stages of qualificationThe FDA’s recently launched ISTAND program, established as a permanent qualification pathway for tools not currently regulated (U.S. Food and Drug Administration, 2026), has now advanced an organ-chip platform (Emulate’s Liver-Chip S1) into formal qualification for predicting drug-induced liver injury, and this could be followed by skin-on-chip platforms (U.S. Food and Drug Administration, 2024). The FDA Modernization Act 2.0 officially eliminated the need for animal testing before human clinical trials, and explicitly included organ chips and microphysiological systems as acceptable substitutes (United States Congress, 2022). In this context, the skin-on-chip platforms are already a particular application. Wound repair was simulated in microfluidic systems under various flow conditions and independent skin irritation assays are shown to be sensitive and specific and present results similar to laboratory models of reconstructed human epidermis (Vilela de Sousa et al., 2023). Concurrently, the EMA has grown more accepting of alternatives to animal testing, such as New Approach Methodologies (NAMs), including organ-on-chip data, and has indicated the use of these tools to model wound-healing efficacy in applications using diabetic wound models (European Medicines Agency, 2025). For modeling wound healing, it is similarly working toward harmonized qualification schemes, representing one of the more significant regulatory science advances of this era (Marx et al., 2020). The direction is clearly toward greater regulatory acceptance, but a defined timeline for formal qualification of skin-specific platforms remains undetermined.

## 6 Ethical and regulatory perspectives

The scientific limitations of animal models and the growing capability of human-relevant alternatives have not developed in isolation from the regulatory and ethical environment. They have developed in active dialogue with it - shaped by requirements that have grown progressively more demanding and by a scientific community that has increasingly recognised the alignment between ethical imperatives and scientific quality.

### 6.1 OECD guidelines and the precedent of validated alternatives

The regulatory validation of RHE models under OECD Test Guidelines 431 and 439 is more than an administrative milestone. It established a proof of concept that has shaped how the entire alternative model field thinks about its future (OECD, 2021). It demonstrated that an *in vitro* system, if sufficiently characterised, standardised, and validated against an agreed performance benchmark, can satisfy the evidentiary requirements of regulatory decision-making in place of an animal test. These models caught on quickly after validation by the cosmetic and pharmaceutical industries, giving rise to a commercial ecology which supports them in their development. The lesson to the wider field of wound healing models is clear: the main obstacle to regulatory uptake is the lack of consensus for validation for complex, multi endpoint models. To validate relevant endpoints for wounds, such as re-epithelialisation kinetics, inflammatory resolution, and angiogenic response, would require finding new validation paradigms that would be appropriate for multidimensional biological endpoints rather than the binary endpoints currently included in OECD guidelines (OECD, 2023). That work is starting, but will need to be a collaborative effort from model developers, regulatory scientists and clinical wound care experts.

### 6.2 The 3Rs as scientific framework

The 3Rs are often discussed as if Replacement, Reduction, and Refinement were three separate imperatives of roughly equal urgency. In the context of wound healing research, they are better understood as a hierarchy of scientific ambition. Refinement - improving the welfare of animals that remain in use - is the minimum acceptable response to ethical concern. Reduction - generating the same information with fewer animals through better experimental design and computational support - is an improvement, but it does not address the underlying problem of translational validity. Replacement - developing human-relevant systems that generate better data without recourse to animal experimentation-is the scientific objective that the 3Rs framework was ultimately designed to motivate (Tannenbaum and Bennett, 2015). The progression of wound healing models described in this review is, in a direct sense, the scientific expression of the Replacement principle. Every progression was not due to ethics only, but to the understanding that the previous system was not providing the correct data and that alternatives would perform better in humans. In this instance, the ethical and the scientific come to a closer alignment than is usually the case.

### 6.3 The realistic trajectory of animal replacement

Complete replacement of animal models in wound healing research is not imminent, and claiming otherwise would not serve the field. To date, no one *in vitro* system or computational model fully replicates the systemic, immunological, vascular and neuroendocrine complexity of wound healing in a living organism, and most endpoints required for the regulatory process for novel therapies for wound healing still depend on animal safety data that cannot be fully replaced by validated alternatives. What is changing - and changing quickly-is the fraction of research questions that can be answered without animals and the quality of answers achieved compared to what is achieved using animal studies. The biological complexity and predictive power of these three-dimensional bioprinted constructs, patient-derived organoids, integrated SoC platforms and AI-guided multi-omics analyses are collectively coming closer to what was not possible 10 years ago (Marx et al., 2020). The realistic near-term path is progressive substitution rather than replacement: animal studies reserved for questions that truly require whole organism biology; human relevant platforms become ever more integral to the preclinical evidence base.

## 7 Challenges, gaps, and future directions

It is important to highlight what the current models are still unable to do, not to minimize their progress, but to focus on the limitations that hinder them from being more translatable. There are three inter-related challenges that are both scientifically important and technically feasible: most engineered systems lack functional vascularisation and immune competence; standardized platforms for wound-on-chip are still missing; and improved integration of advanced biomaterials that can replicate the dynamic extracellular matrix (ECM) environment of the wound.

### 7.1 Vascularisation and immune competence

In wound healing, the vasculature does not simply carry out its function, it is a player. The dynamic blood flow controls recruitment of inflammatory cells, oxygen and metabolite gradients, angiogenic signalling and physical forces which guide repair and cell behaviour throughout. This is not captured well by the existing engineered wound models. Diffusion-limited transport in 3D constructs and organoids is not equivalent to perfused circulation and the barrier-forming complexity and haemodynamic responsiveness of current SoC devices does not match that of native microvasculature (Abaci et al., 2016; Bhatia and Ingber, 2014). The immune gap is also considerable: macrophages, neutrophils, and T lymphocytes are not fully represented or missing from many platforms, although the control of inflammation over time is the key factor in determining if wounds heal or become chronic (Rognoni and Watt, 2018). Addressing both gaps in a single integrated platform - vascularised, immune-competent, perfused-is the defining technical challenge of the next-generation of wound healing models. The tools to attempt it - 3D bioprinting of vascular networks, iPSC-derived immune cell differentiation, advanced microfluidic integration - exist and are being combined; the question is whether the resulting systems can be made sufficiently reproducible and accessible to serve as routine research platforms.

### 7.2 Standardisation of wound-on-chip platforms

The scientific credibility of any experimental system depends on reproducibility across laboratories, which in turn requires standardization. Current wound-on-chip models vary substantially in device geometry, membrane composition, cell source and passage number, culture medium formulation, wound induction method, and analytical endpoints, variability that makes it nearly impossible to compare results across studies or to build a coherent evidence base for regulatory purposes (Marx et al., 2020). Wound induction methods alone, like mechanical scratching, laser ablation, and chemical disruption, produce wounds of different depth, geometry, and cellular response profile, and the choice between them is rarely justified against a biological rationale. Without shared protocols, benchmarks, and validation criteria, individual studies have limited value. In fact, the overall scientific impact of these scattered efforts is less than what could be achieved if everyone used a single, well-validated protocol. Developing a harmonized international standard for wound-on-chip systems, as the OECD did for RHE models, will require bringing together model developers, clinicians, regulatory scientists, and standards organizations from the start.

Three things are needed to make real progress. First, all studies should use a minimum reporting dataset that includes details like the cell or tissue source and passage number, the wound induction method with its physical parameters (such as wound depth, width, or flow rate), and clearly defined functional endpoints (like re-epithelialization rate, wound closure percentage, and measurement time points). Second, there should be an inter-laboratory validation process, set up as a ring trial where different labs use the same reference compounds and score outcomes blindly. This would help define the reproducibility of the results before considering regulatory approval, following standards such as ASTM International’s MPS terminology standard and the ISO 22916:2022, which established interoperability requirements for organ-on-chip device dimensions, connections, and classification. Third, standardization efforts should involve model developers, clinicians, regulatory scientists, and standards organizations (such as ASTM, ISO, and CEN-CENELEC, which has coordinated cross-sector workshops to build a roadmap toward formal OoC standardisation, ‘Putting Science into Standards’ workshop (Piergiovanni et al., 2021)) from the very beginning, not just as reviewers after the technology is developed.

### 7.3 Integration of advanced biomaterials

The ECM of a healing wound is not a stable scaffold -it is a dynamically remodelling, biochemically instructive microenvironment whose composition, stiffness, and signalling properties change across the phases of repair and actively regulate cell behaviour at each stage. Most current wound models use static collagen gels or fibrin matrices that approximate the structural properties of provisional matrix but do not replicate its instructive dynamics. Smart and functionalised hydrogels - stimuli-responsive matrices, growth factor-loaded scaffolds, nanoparticle-embedded systems - are increasingly available as research tools (Peppas et al., 2006), but their integration into structured wound healing model platforms has been limited. This is a missed opportunity in both directions: advanced biomaterials would enhance the biological realism of wound models, and wound models provide the most relevant platform for evaluating advanced biomaterial performance. SoC systems incorporating perfusion alongside functionalised ECM scaffolds can, in principle, model drug release kinetics, cellular responses to matrix cues, and tissue regeneration simultaneously under physiologically relevant dynamic conditions - a capability that would substantially improve the predictive value of preclinical biomaterial evaluation. The construction of these types of hybrid devices, in which biomaterials, microfluidics and multicellular complexity are merged in a single platform, is the most promising trajectory in the field, and the recent strides in multi-material bioprinting are making these technical challenges more manageable (Lee et al., 2026).

## 8 Emerging research frontiers

**FIGURE 4:**
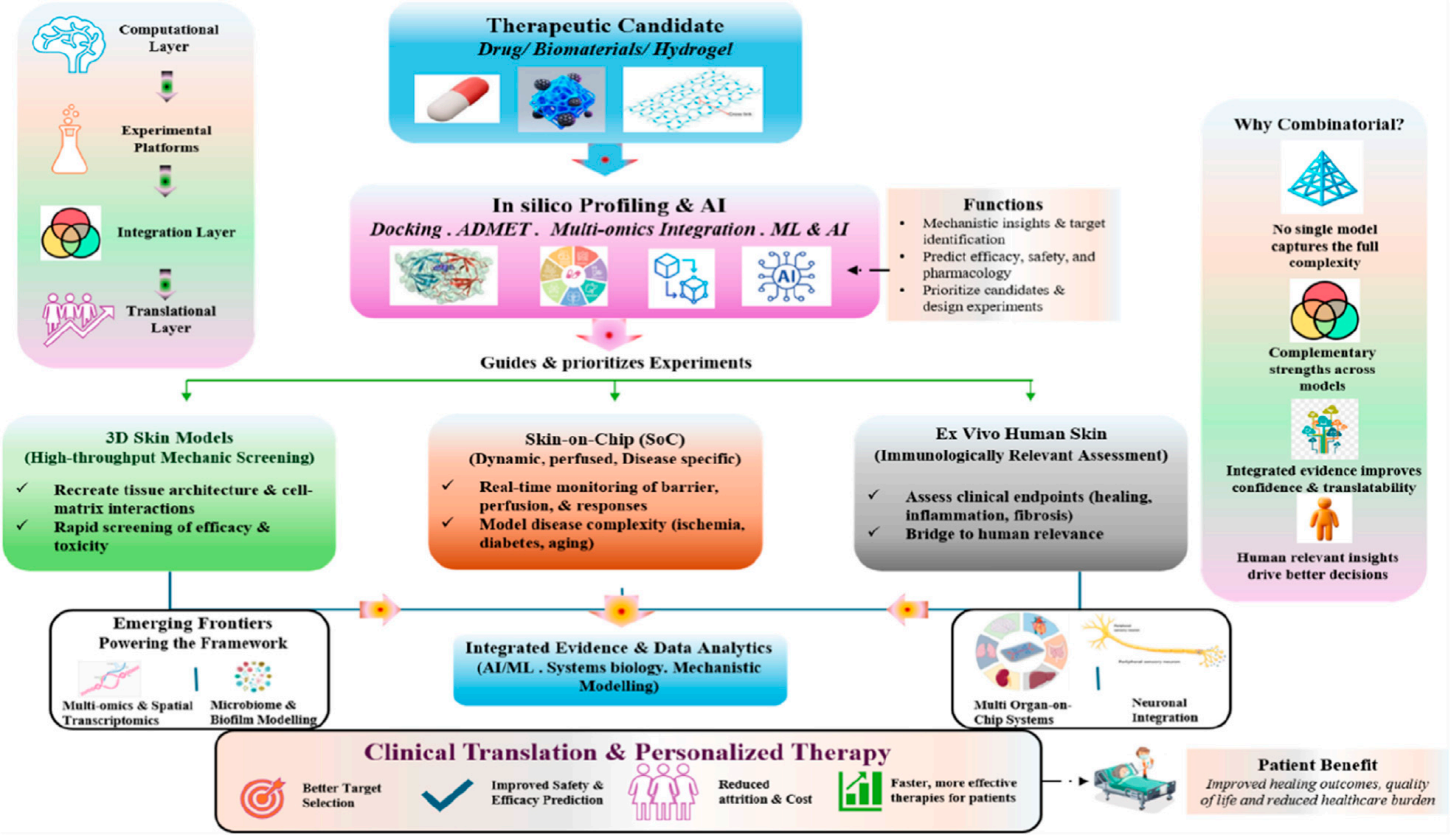
The image Illustrates Combinatorial Tier System Showing How a Therapeutic Candidate would move from *in-silico* to SoC validation to *Ex-vivo* to Clinical Translation.

The model platforms mentioned above represent the state of the art in human-relevant wound healing studies. Over the next decade, developments in molecular profiling, microbiology, systems engineering, and computational science will have a significant impact on these frontiers. The advances described below are not peripheral, but instead focus on aspects of the field that are not sufficiently modelled by existing approaches and will have a direct impact on the most persistent and clinically important wound healing challenges (See Figure 4).

### 8.1 Multi-omics integration and spatial transcriptomics

To date, scRNA-seq has yielded a new perspective to the cellular mechanisms underlying wound healing, revealing transcriptional diversity within subsets of macrophages, fibroblast lineages and keratinocytes, which could not be distinguished by bulk tissue analysis (Berthiaume Fox et al., 2026). That what we had previously described as a ‘wound macrophage’ is actually a mixture of distinct cell states, with different temporal dynamics and association with healing outcome, is not a ‘refinement’ of the nomenclature used in the academic world, it is a paradigm shift in the therapeutic target landscape for chronic wound intervention. Spatial transcriptomics further extends this resolution to the tissue dimension and allows the interrogation of the spatially resolved signalling gradients, immune infiltration patterns, and ECM remodelling zones that would be lost by single-cell dissociation. Integrating multi-omics data with SoC platforms and computational models creates a route toward wound healing atlases that link molecular events to tissue-level outcomes with precision that was not available at any previous point in the field’s history- and that provides the benchmarking data needed to assess how closely any experimental model actually recapitulates native wound biology.

### 8.2 Skin microbiome and biofilm modelling

The wound microbiome is, at present, almost entirely absent from experimental wound healing models, and the gap between laboratory practice and clinical reality this creates is substantial. Chronic wounds are not sterile, they are colonised environments where the composition of the microbial community, the interaction between different microorganisms and the structure of the formed biofilm plays a key role in determining if the wound becomes infected or remains sterile (Flynn et al., 2023). *Staphylococcus aureus* and *Pseudomonas aeruginosa* in biofilm configuration are not merely harder to kill than their planktonic equivalents - they actively remodel the host immune environment, inducing chronic inflammatory signalling whilst evading phagocytic clearance. Modelling this in a human-relevant tissue context requires *ex vivo* or SoC systems capable of sustaining defined microbial communities under controlled conditions -a technical challenge that is beginning to be addressed but has not yet been standardised Such would not only enhance the biological fidelity of chronic wound models, it also would provide the most relevant preclinical platform to test antimicrobial wound dressings, bacteriophage therapy and microbiome-targeted therapeutics.

### 8.3 Multi-organ-on-a-chip and systemic modelling

Wound healing does not occur in an isolated skin compartment, it occurs in the context of whole-body physiology, and that context matters in ways that single-tissue models cannot capture. A drug metabolizing organ, such as the liver, determines the concentration and form of systemically delivered therapeutics at the wound; an organ with renal clearance regulates the pharmacokinetics of the duration of a drug; immune cells flowing through the blood from remote organs contribute to inflammatory/reparative events; and in diabetes, organs distant from the wound bed contribute to metabolic/vascular dysfunction that underlies chronic wound pathology. Multi-organ-on-a-chip platforms representing the skin, liver, kidney, immune, and vascular circuits are connected via common fluidic networks to address the systemic dimensions that cannot be captured by any single-organ model (Bhatia and Ingber, 2014). For wound therapeutic evaluation particularly for systemically delivered agents, the gap between single-organ SoC data and multi-organ data is not marginal; it is the gap between knowing what a drug does in a skin construct and knowing what it does in a body.

### 8.4 Artificial intelligence and machine learning

As the complexity of the biological dimension of advanced wound healing models rises, the data generated becomes more dimensional and voluminous to deal with using traditional means. AI and machine learning are not just capabilities that are optional additions to this research landscape, they are becoming must-have infrastructure. The use of AI and machine learning is no longer a luxury in this research scenario but a necessity. Wound images, either clinical photographs, histological sections or real-time data from microfluidic experiments, can be used to measure wound parameters by deep learning algorithms with greater accuracy and objectivity than can be done by manual assessment at scale (Berthiaume Fox et al., 2026). Applied to high-content imaging on SoC platforms, AI-assisted analysis provides interpretability of phenotypic features (cell morphology, migration trajectories, vascular network topology, ECM organisation) that are manually intractable and allows knowledge of the mechanism to be gained from the datasets that would otherwise be analytically opaque. Pre-trained AI models can analyse integrated multi-omics wound healing datasets to uncover markers of healing failure, stratify patients for clinical trial design, and provide predictions of the healing trajectory for each individual patient, opening the door to the promise of precision wound medicine. The quality of these tools depends on the quality and scale of the datasets used to build them, and the field would benefit substantially from coordinated, open-access wound healing data infrastructure that does not currently exist.

### 8.5 Neuro-immune crosstalk

**FIGURE 5:**
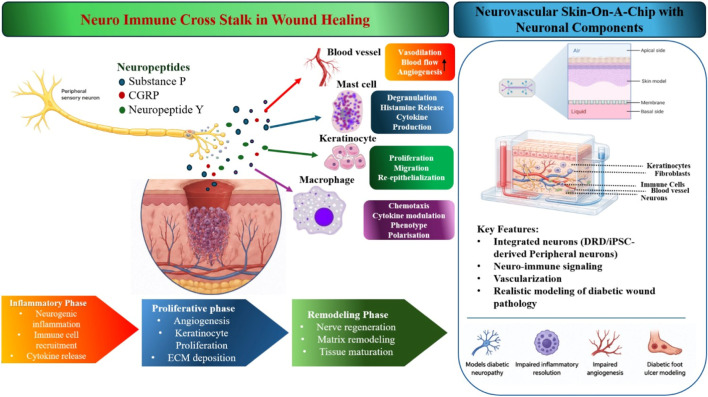
The image illustrates neuro-immune crosstalk during wound healing in the first part, and depicts a neurovascular skin-on-a-chip model incorporating neuronal components in the second part.

The peripheral nervous system is a regulator of wound healing that has received far less experimental attention than its clinical significance warrants. These peptides, namely, substance P, CGRP and neuropeptide Y, control vascular tone, mast cell activation, keratinocyte proliferation and macrophage function and have direct relevance to the inflammatory and proliferative stages of repair, which occur at the periphery (Berthiaume Fox et al., 2026). Diabetic peripheral neuropathy, the progressive loss of sensory and autonomic innervation that affects the majority of patients with long-standing diabetes which disrupts this neuro-immune signalling in the wound environment, impairing inflammatory resolution and angiogenic responses in ways that are not reproduced by any current wound model (See Figure 5). In skin-on-a-chip and organoid platforms, the lack of neuronal components is not just a lack in completeness; it is the fact that these systems are not able to correctly model one of the main pathological processes that leads to the formation of diabetic foot ulcers. The inclusion of dorsal root ganglion neurons or iPSC derived peripheral neurons is technically possible and has been shown in proof-of-concept studies and the development of standardised and reproducible neurovascular skin chip systems is a research priority with clear clinical relevance.

## 9 Discussion and conclusion

In fact, the history of wound healing research is, to a large extent, the history of the more complicated and sophisticated attempts to create experimental systems that recreate what the body does and then the honest reckoning of how far each generation of systems falls. Two-dimensional cell cultures set the stage for molecular definitions of wound cell biology, 3D constructs added tissue architecture, *ex vivo* preparations added immunological competence, organoids added self-organised developmental complexity and skin-on-a-chip platforms added dynamic, perfused cell biology, a system that cannot be recreated in static settings. All of the advances were real, and all were needed, but none enough.

The core claim of this review is that sufficiency is the wrong standard by which to judge preclinical wound healing models. No single model will replicate the systemic, immunological, vascular, microbial, and neuroendocrine complexity of human wound healing - not because this is impossible in principle, but because clinical and biological questions that are relevant to wound care are numerous and diverse, thereby ruling out the possibility of answering them with only one experimental system. The computational layer functions as the primary integrator, producing mechanistic and predictive insights that guide experimental design, *in silico* modelling, and artificial intelligence analysis. This layer interfaces with three-dimensional systems for high-throughput mechanistic screening, *ex vivo* preparations for immunologically relevant endpoint assessment, and system-on-chip platforms for dynamic, disease-specific therapeutic evaluation. This, deliberately applied in layers and in the right order according to the biological question to be addressed, is a translational wound healing research program.

The emerging frontiers discussed in Section 8 are not just side-shows but the next architectural level in this paradigm. Multi-omics and spatial transcriptomics offer the molecular benchmarking that brings a new level of resolution needed to verify model accuracy and to uncover therapeutic targets. Microbiome and biofilm modelling will close the largest single gap between laboratory wound models and the clinical wounds they are meant to represent. Multi-organ-on-a-chip systems will extend human-relevant wound pharmacology to the systemic context that single-tissue models cannot access. AI and machine learning will make the data generated by these increasingly complex systems analytically tractable and clinically actionable. Neuronal integration will, for the first time, make it possible to model the neuro-immune dysregulation that underlies diabetic wound pathophysiology in a human-relevant tissue system.

Research on wound healing has been fragmented across various disciplines and the translational gap in this field cannot be bridged by any one discipline working alone. The developed models are in the areas of cell biology, materials science, microengineering, and immunology, with clinical wound care and regulatory science expertise required to validate these models. Greater integration of institutions with more flexible coordination and funding systems will be needed to achieve broader adoption. The lag between developing new drugs in the laboratory and making them or their components available for clinical use has been longstanding. Tools that can help meaningfully close this divide are more readily available than ever before. All that are left are the disciplines and institutional commitment needed to put them together in action.
